# Toward three-dimensionally ordered nanoporous graphene materials: template synthesis, structure, and applications

**DOI:** 10.1039/d3sc05022j

**Published:** 2023-12-26

**Authors:** Masanori Yamamoto, Shunsuke Goto, Rui Tang, Kaoru Yamazaki

**Affiliations:** a Department of Chemical Science and Engineering, Tokyo Institute of Technology Ookayama 2-12-1 Meguro Tokyo 152-8550 Japan yamamoto@mol-chem.com; b Institute of Multidisciplinary Research for Advanced Materials, Tohoku University 2-1-1 Katahira, Aoba Sendai 980-8577 Japan; c RIKEN Center for Advanced Photonics, RIKEN 2-1 Hirosawa Wako Saitama 351-0198 Japan kaoru.yamazaki@riken.jp; d Institute for Materials Research, Tohoku University 2-1-1 Katahira, Aoba Sendai 980-8577 Japan

## Abstract

Precise template synthesis will realize three-dimensionally ordered nanoporous graphenes (NPGs) with a spatially controlled seamless graphene structure and fewer edges. These structural features result in superelastic nature, high electrochemical stability, high electrical conductivity, and fast diffusion of gases and ions at the same time. Such innovative 3D graphene materials are conducive to solving energy-related issues for a better future. To further improve the attractive properties of NPGs, we review the template synthesis and its mechanism by chemical vapor deposition of hydrocarbons, analysis of the nanoporous graphene structure, and applications in electrochemical and mechanical devices.

## Abstract 2

Precise template synthesis will realize three-dimensionally ordered nanoporous graphenes (NPGs) with a spatially controlled seamless graphene structure and fewer edges.

## Graphical abstract

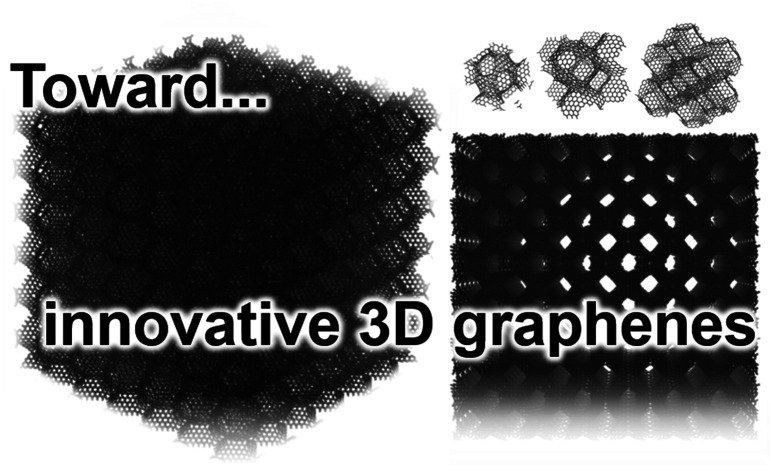

## 1. Introduction

Carbon materials have industrial applications owing to their excellent features such as electrical conductivity, chemical and thermal stability, light weight, and low cost of preparation.^1^ Nevertheless, the actual structures of carbon materials have not been well documented until recently, except for their chemical composition. Recent developments in analytical technologies for probing the structure of carbon materials, such as transmission electron microscopy,^2–4^ Raman spectroscopy,^5–7^ and high-sensitivity temperature-programmed desorption methods,^8,9^ have enabled understanding of the actual three-dimensional (3D) structure of carbon materials at the molecular and atomic levels.

With the developed tools for nanoscale analysis, the synthesis and understanding of nanostructured carbon materials have further expanded their fields and applications. Various synthesis methods have been investigated with the aid of advanced nanostructural analysis for nanostructured carbon materials, including arc discharge,^10^ template carbonization,^2,11^ conversion of graphene oxides^12,13^ to their reduced analogs,^13,14^ fine organic synthesis,^15,16^ topochemical pyrolysis,^17–19^ and chemical vapor deposition (CVD).^20–24^ Consequently, many advanced carbon materials, including carbon fibers, carbon nanotubes (CNTs), graphenes, structural graphite, and carbon foams have been developed with improved physicochemical properties, and they are growing at a compound average growth rate (CAGR) of ∼6% with an annual global market of 3 billion USD in 2015.^25^ They are extensively used as adsorbents,^26^ catalysts,^27–33^ catalyst supports,^34^ conductive additives,^35–37^ and anode materials^38^ in batteries, supercapacitors,^39,40^ polymer-electrolyte fuel cells (PEFCs),^41^ and photothermal conversion.^42^

In this context, the chemical science of two-dimensional (2D) graphene and its zero-dimensional (0D) and one-dimensional (1D) analogs has been extensively studied in recent decades. Graphene,^43^ a 2D-carbon material, was originally prepared by exfoliation, and its synthesis, analysis, and applications have been extensively investigated.^44–48^ Single-plate graphene materials have high electrical conductivity and high thermal/chemical stability; therefore, they are suitable for practical applications in energy-related devices. A class of 1D-graphene, carbon nanotubes^49,50^ and cylindrical analogs,^51^ has also been investigated for electrochemical and catalysis applications owing to their high electrical conductivity.^52^

A challenging aspect is the synthesis of the corresponding 3D graphene materials^53–55^ from a 2D scaffold.^13^ Reducing the dimension of 2D graphene can be used to develop 3D graphene; the 0D analog of the fullerene family,^56^ a good electron acceptor,^57^ can be used as a building block to develop 3D-analogs.^58^ Catalytic carbonization has also been successfully applied to prepare 3D graphene architecture.^23,29,59^ However, the precise synthesis of 3D-ordered graphene materials with well-defined negatively curved 3D networks^60,61^ remains an emerging topic since British scientists predicted imaginary carbon frameworks with 8-membered rings in hexagons to achieve a 3D structure with low strain.^53^ These materials are highly symmetric and possess highly ordered nanostructures that provide various fascinating functions (*vide infra*). For example, material surfaces for the adsorption of gases should be as homogeneous as possible with respect to the structure and chemical composition^62^ for better and faster reactions, and imaginary 3D-ordered carbon frameworks should meet these criteria. The novel electronic nature of these frameworks makes them appealing for various applications.

**Fig. 1:**
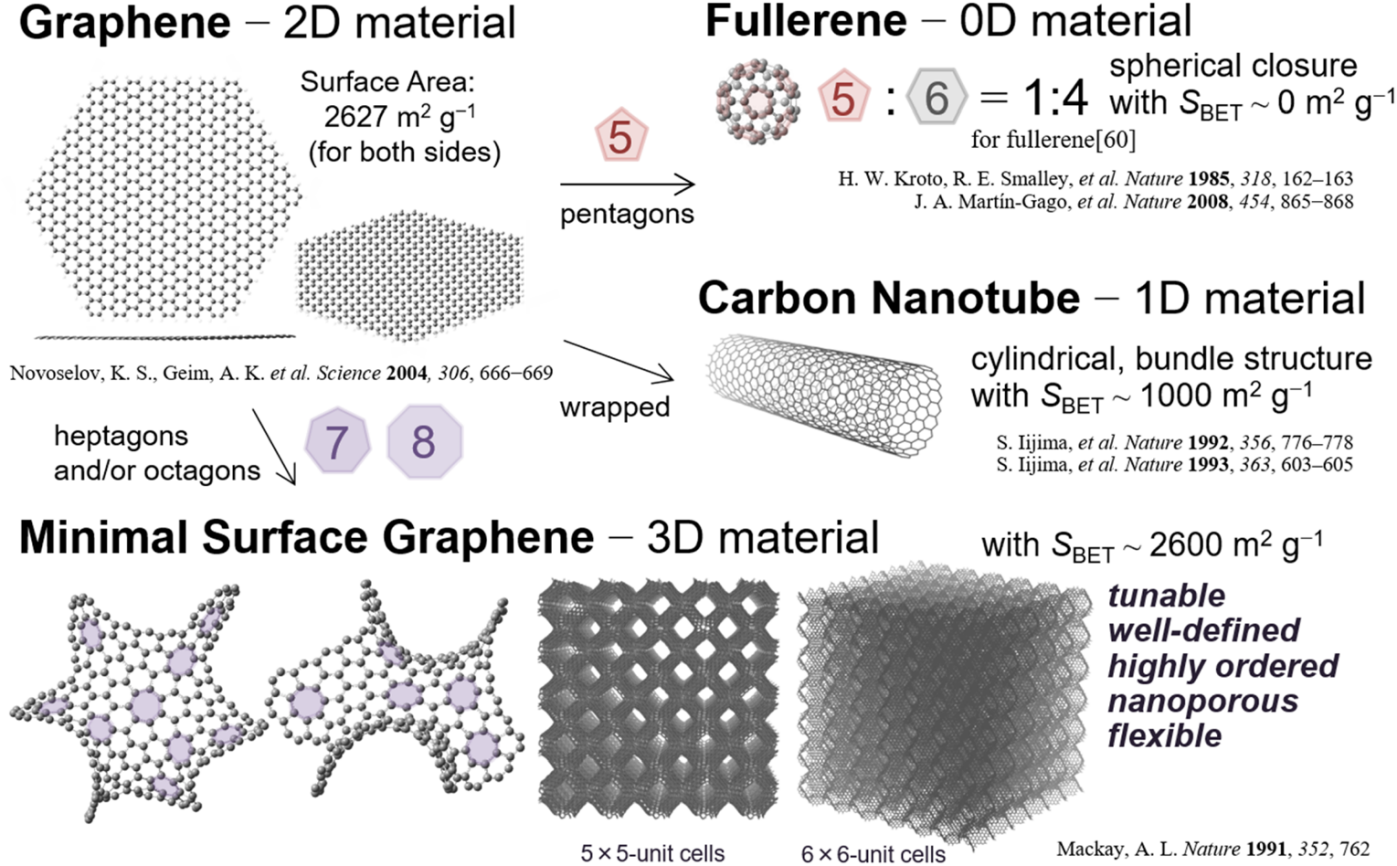
Schematic of structurally well-defined graphene analogs with various dimensions of materials, including 3D graphene materials.^59^ Gravimetric surface area of [60]fullerene is derived from the literature.^70^

In this review, we provide an overview of recent progress in the synthesis, structural characterization, and application of electrochemical and mechanical devices of 3D-ordered porous graphene material nanostructures. The remainder of this paper is organized as follows: in Section 2, we discuss typical synthetic procedures using templating carbonization on nanostructured precursor materials, such as zeolite and alumina. We then focus on the reaction mechanism of the carbon growth mechanism of CH_4_-CVD on alumina and the structure of the resultant 3D graphene, directed toward achieving a periodically ordered graphene nanostructure (Fig. 1). In Section 3, we provide an overview of how the 3D continuous nature of porous graphene drives its potential applications in electric double-layer capacitors, next-generation Li-ion batteries, and refrigeration devices based on mechanical force. In Section 4, we summarize this review and describe our outlook for further fundamental and application studies on 3D-ordered graphenes in the near future.

## 2. Synthesis and structure of templated carbons

A template method (*alias dictus* nanocasting)^63^ has been explored for the synthesis of well-defined nanostructured materials. The chemical and physical properties of the obtained nanomaterials can be tuned by using the synthesis procedure as well as the precursor materials,^2^ and CVD is a powerful method to precisely control nanostructures at the atomic level.^64^ For example, Ito and co-workers reported sophisticated macroporous (>50 nm) 3D graphene materials using CVD of benzene on a porous Ni template.^23^ The material maintains the electronic nature of pristine 2D graphene, and its analogs with structural defects, including substitutional defects and adatoms of nitrogen and sulfur, work efficiently as catalysts.^28,29,65^ owing to their high electrical conductivity. Recently, the effects of structural topology^66,67^ and mechanical^68^ and electronic properties^69^ have also been well studied. Thus, these materials are excellent candidates for developing ideal 3D graphene materials. However, we recognized that a 3D single-layered graphene architecture with a large pore size and high surface area (>2000 m^2^ g^−1^) sometimes collapses and reduces the structural regularity of the grown carbons during the template removal process using aqueous acids, as previously reported,^22^ leading to stacked carbon layers. Hereafter, we focus on porous graphene analogs with a narrow pore distribution (<20 nm).

### 2.1 Zeolite-templated carbon (ZTC)

Zeolites are a class of inorganic materials composed of SiO_2_ and Al_2_O_3_ (aluminosilicates) with alkali metals as countercations. They exhibit high surface areas with highly ordered micropores and are widely used in catalysts.^71–76^ The catalytic ability and structural regularity of zeolites^77,78^ are enticing features for their use as templates for synthesizing nanostructured carbon materials. In 2000, Kyotani and co-workers reported the synthesis of zeolite-templated carbon (ZTC) using Y zeolite (faujasite, FAU) as the template material.^11^ The studies used Y zeolite with a porous diamond-like framework of spherical cavities (supercage, ∼1.3 nm in diameter):^79^ ZTC was prepared by transcription of the structural information of zeolites. A composite of furfuryl alcohol and Y zeolite powder was heated, followed by treatment with propylene at 600–800 °C for CVD to achieve the ordered porous structure of single-walled nanographene derivatives.^79^ Thermal annealing at 900 °C for 3 h ensures a more robust 3D structure.^79,80^ After CVD, the resulting material was washed with HF and HCl solutions to obtain the ZTCs. This procedure allowed the ZTC to retain the highly ordered microstructure of the zeolite with carbon architecture.

Ordered microporous structures provide a high surface area, large micropore volume, and fast diffusion rate of gases and electrolytes despite the narrow pore width. These features are suitable for gas physisorption^81^ and electric double-layer capacitor^82^ applications. The XRD pattern of the carbon material synthesized using Y zeolite as a template shows a peak at 2*θ* = 6.26°, which corresponds to the 111 diffraction of the parent Y zeolite at 2*θ* = 6.19°. This XRD pattern indicates that the original ordered structure of Y zeolite is maintained in the carbon material. The sharp diffraction peak indicates that the carbon material has excellent long-range order with a periodicity of 1.41 nm, which is the same as the 111 spacing of the Y zeolite. The adsorption isotherm shows a sharp increase in N_2_ adsorption as *P*/*P*_0_ increases in the low-pressure region and quickly reaches a plateau, indicating that the microporous structure exhibits a minimal number of mesopores or macropores. Both sides of the single-walled graphene-like ZTC surface can act as solid–liquid or solid–gas interfaces. In addition, edges also contribute to the surface area of ZTC, which reaches a gravimetric surface area (*S*_g_) of >3000 m^2^ g^−1^. This feature enables efficient uptake of CH_4_ (ref. 26) and H_2_.^81^

Porous carbon materials with the structural 3D regularity of the original parent material can also be synthesized by carbonization using other zeolites and inorganic nanomaterials. Acidity, as confirmed by pyridine-IR spectroscopy, plays an important role in controlling the quality of ZTCs.^83^ N-Doped^84,85^ and B/N-doped^85^ ordered carbon materials have also been prepared using zeolite-templated methods. They demonstrate the feasibility of the templating method for preparing ordered carbonaceous materials.

### 2.2 Nanoporous graphene (NPG)

#### 2.2.1 Synthesis

The CVDs of gaseous unsaturated carbon sources with C≡C triple bonds, such as acetylene, and C=C double bonds, such as propylene, have been investigated to prepare templated carbons.^11,87^ The resultant periodically arranged microstructured carbon cages enable efficient diffusion of gases and electrolytes.^23^ Typically incompatible high surface area, fast diffusion of gases and electrolytes, electrical conductivity, and structural flexibility are realized using the templated method. However, control of uniform single-layer graphene growth is difficult, with significant amounts of fragile oxygen-rich edge sites being formed to reduce the chemical and electrochemical stability of the obtained carbon materials.

By contrast, aliphatic hydrocarbons such as CH_4_ are relatively inert for carbon deposition reactions.^88^ It is expected that the efficient catalytic deposition of CH_4_ on an ordered 3D template can achieve ideal 3D graphene with tailored micro-porosity.

**Fig. 2:**
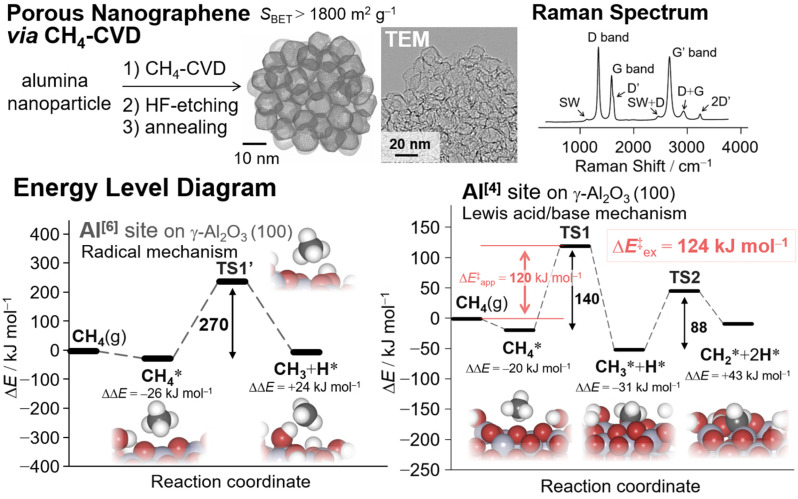
(Top) Schematic of the synthesis of nanoporous graphene materials *via* CH_4_-CVD^59^ on Al_2_O_3_ nanoparticles and their analysis^86^ (reprinted with permission from ref. 86. Copyright 2023 Royal Society of Chemistry). (Bottom) Energy level diagrams of the initial CH_4_ activation on the Al^[6]^ site and Al^[4]^ site of the γ-Al_2_O_3_ (100) surface during CH_4_-CVD for the synthesis of NPGs.^59^

Nanosized γ-alumina is a promising template material for synthesizing nanoporous graphene (NPG) materials since it is one of the most efficient catalysts for catalytic C–H activation.^89^ CH_4_-CVD takes place on alumina nanoparticles (ANPs) with the initial associative dissociation of CH_4_ as the kinetic bottleneck,^59^ and this is followed by acid etching and annealing^86,90–93^ to give NPGs as minimally stacked porous graphene analogs (Fig. 2). The resultant NPGs have a high surface area (>1800 m^2^ g^−1^), high electric conductivity, elastic properties, and a sponge-like mesoporous framework.

The use of inert CH_4_ enabled us to selectively synthesize single-layered porous graphene materials. Density functional theory (DFT) calculations coupled with experimental kinetic analysis using thermogravimetry (TG) techniques revealed that the dehydration and subsequent surface activation of γ-ANPs by CH_4_ are crucial in controlling surface chemistry.^59^ According to the DFT calculations, the original octahedral site (Al^[6]^) coordinated with six atoms of surrounding oxides affords a radical mechanism in the initial CH_4_ activation, with an activation energy (Δ*E*^‡^) of 270 kJ mol^−1^. Further elimination of surface oxides eventually leads to more reactive tetrahedral sites (Al^[4]^). The surface with oxygen vacancies provides a favorable reaction pathway for proton transfer (PT) reactions *via* the Lewis acid/base mechanism, with a calculated apparent Δ*E*^‡^ of 120 kJ mol^−1^. This theoretical value agrees well with the experimental value of 124 kJ mol^−1^ obtained from the Arrhenius plot using the TG technique.^59^ This PT step is the kinetic bottleneck for the entire reaction. Subsequent PT results in the formation of surface methylene species 
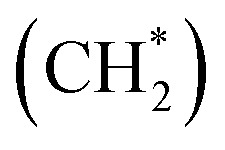
. DFT calculations also demonstrated that further carbon chain growth to form heavier aliphatic hydrocarbons by adding or inserting 
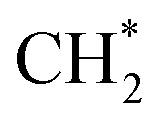
 species is thermodynamically and kinetically feasible.^94^ θ-ANPs are also applicable as template materials.^86,92^

**Fig. 3:**
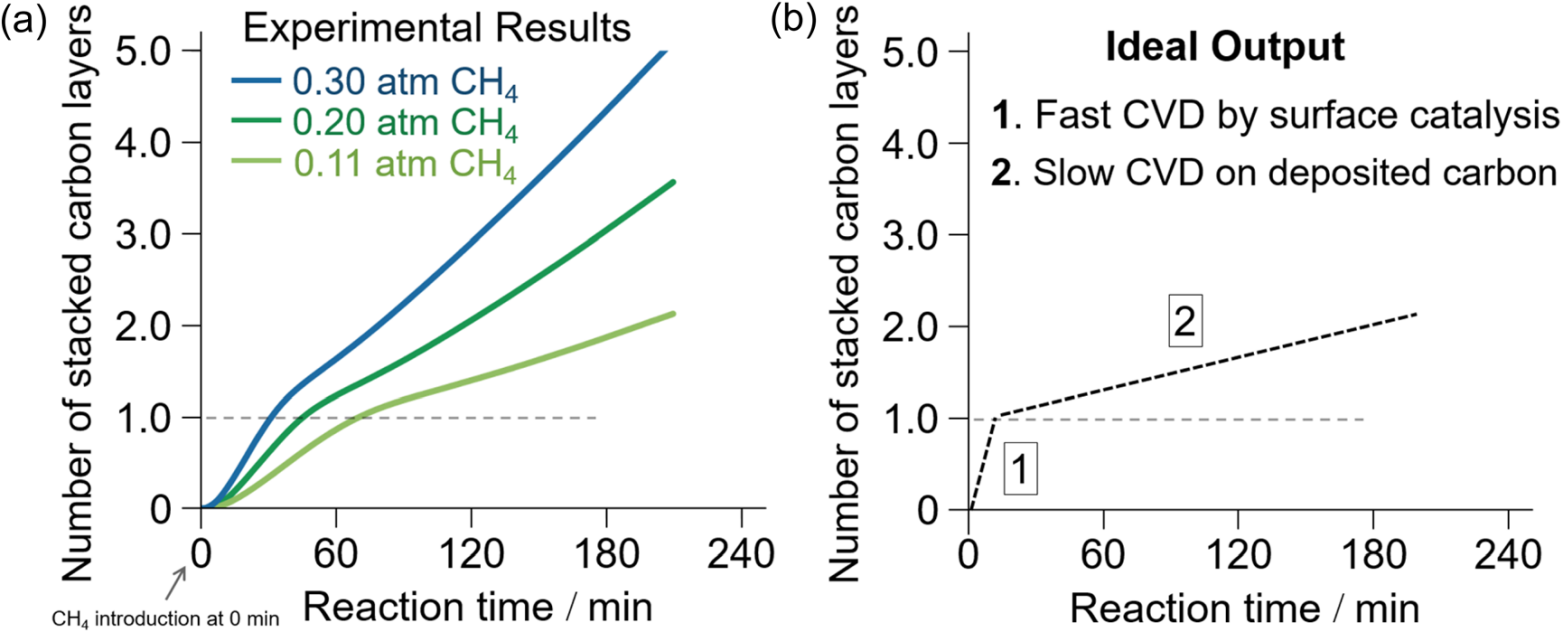
(a) TGA of CH_4_-CVD on γ-Al_2_O_3_ nanoparticles at 900 °C with different partial pressures of CH_4_.^59^ (b) Expected ideal output of the TGA of CH_4_-CVD with enhanced surface catalysis for selective synthesis of single-layered porous graphene materials with improved integrity of “graphene nature”.

To improve the efficiency of the synthesis and quality of the resultant NPGs and achieve ideal 3D graphene materials, it is essential to find new templates that realize (i) efficient catalytic activation of CH_4_ at lower reaction temperatures, (ii) better structural regularity of the resultant NPGs, and (iii) easy removal of the templates after the CVD process. CH_4_-CVD on nanostructured oxides, including γ-ANPs and zeolites, requires high temperatures above 800 °C for a sufficient rate of carbonization.^59^ Such a high temperature can easily damage the structural order over time.^101^Fig. 3a shows the dependence of CH_4_ partial pressure on the rate of CH_4_-CVD carbonization on γ-ANPs. We found that the rate of carbonization decreased when the reaction reached the single-layer carbonization on γ-ANPs. Faster carbonization through better surface catalysis, as shown in Fig. 3b-1, is required to lower the reaction temperature and improve selectivity. Screening of catalysts using thermogravimetric analysis (TGA) is a promising approach.

The economical preparation of carbon materials is also important for practical applications.^102^ Template materials investigated to date, such as nanostructured silica,^103,104^ γ-ANPs,^59,86^ θ-ANPs,^92^ and zeolite,^11^ require the use of hazardous HF aqueous solutions for their removal after CVD, which makes the synthetic process tedious. For practical industrial applications, HF should be replaced with the less hazardous HCl. Catalytically active MgO^105–107^ is a promising template material^24,108,109^ that does not require HF during template etching. Investigating the dissolution of ANPs and zeolites in benign and safe ways is crucial for the practical application of NPGs.

#### 2.2.2 Structure

NPGs exhibit high surface area and very few edge sites. Table 1 summarizes the physicochemical properties of representative carbon materials. Another important feature of meso-structured elastic carbons is their highly flexible carbon framework. The bulk modulus of NPG is less than 1 GPa, and this value is a magnitude smaller than those of typical “soft” materials such as zeolite (14 GPa) and the relatively soft class of metal–organic-frameworks (7.7 GPa).^99^

**Table 1:** Summary of the physicochemical properties of materials

Materials	*S*_BET_/m^2^ g^−1^	*d*_p,BJH_/nm	*V*_p_/mL g^−1^			*σ*/S cm^−1^	*N*_edge_ c/mmol g^−1^	*K* d
			Total	Micro^a^	Meso^b^			
NPC (this work)	2300	9.2	5.30	0.85	4.5	0.52^e^ (powder)	3.1	0.9 GPa
NPG (this work)	1910	9.0	4.10	0.70	3.4	0.97^e^ (powd.)^92^–18 (sheet)^95^	0.16	0.3 GPa
Macroporous graphene^23^	∼1260 (ref. 42)	25 (ref. 69)–200 (ref. 23)	—	—	—	∼10^6^ (ref. 23 and 67)	—	<0.1 GPa (ref. 68)
Graphene	(2627)^f^	—	—	—	—	(10^6^)	—	1.0–2.3 TPa (ref. 96–98)
SWCNTs	1300 (ref. 95)	—	3.0 (ref. 95)	—	—	6.8 (ref. 95)	0.48 (ref. 95)	(1.2 GPa)^99^
MSC30	2841	2.3	1.6	0.92	0.7	1.29^e^	2.3 (ref. 100)	1.57 GPa

a Obtained from the DA plot.

b *V*_meso_ = *V*_total_ − *V*_micro_.

c Concentration of edge sites (*N*_edge_) calculated by using TPD.

d Bulk moduli (*K*), or otherwise, the calculated Young's modulus in parentheses.

e At an applied pressure of 90 MPa.

f Ideal values are shown in parentheses.

The surface area of NPGs mainly comprises the basal plane of single-layer graphene extended over the 3D nanospace instead of the edge sites.^92^ The surface area of NPGs obtained by using a γ-ANP template reached 1800–2300 m^2^ g^−1^, and this lies between that of single-layered graphene (2627 m^2^ g^−1^) and stacked bilayer graphene (1314 m^2^ g^−1^).^59^ Temperature-programmed desorption (TPD)^8^ of NPGs was investigated, and it showed much fewer edge sites on NPGs when compared with typical activated carbons and carbon black.^86,92^ The electrochemical stability of NPGs also confirms that the above-mentioned structure has fewer terminal edge sites.^92^

The Raman spectra of NPGs indicate that they consist mostly of hexagonal aromatic rings, but some pentagons and heptagons exist, which introduce curvature to the NPGs in their grain boundaries.^86^ An intense G band at approximately 1587 cm^−1^, as in typical graphene^110,111^ (Fig. 2), coupled with a high specific surface area (*S*_BET_ ∼2000 m^2^ g^−1^) and a weak 002 diffraction in the XRD pattern indicate the presence of a single-layered graphene structure in NPGs.^86^ The red-shifted G′ band at ∼2670 cm^−1^, compared with that of typical graphite,^110,111^ corroborates the presence of a single-walled graphene structure.^44,110^ The red-shifted G′ band may also reflect the value of Young's moduli^96^ by the introduction of curvature. In general, a relatively sharp D band (Fig. 2) in the Raman spectra can be mainly attributed to edges,^6,7^ but TPD analysis indicates a very low occurrence of edges.^86,92^ Therefore, the prominent D band of the NPGs is mainly attributed to in-plane disorder instead of edges.

The type of disorder can be qualitatively analyzed using the intensity ratio between the D and D′ bands, *I*_D_/*I*_D′_.^112^ The *I*_D_/*I*_D′_ of NPGs is *ca.* 5. This indicates that these NPGs have both vacancies and grain boundaries since the *I*_D_/*I*_D′_ values lie between those of graphene dominantly with vacancies (∼7) and grain boundaries (∼3.5).^112^ The shoulder peak at ∼1150 cm^−1^ in the Raman spectra proves the existence of Stone–Wales (SW) defects,^5,112–115^ while the small peak at ∼2460 cm^−1^ can be attributed to the SW + D band.

The mean distance between the defects *R* can be related to the intensity ratio of the D and G bands, *I*_D_/*I*_G_(*R*):^116^1
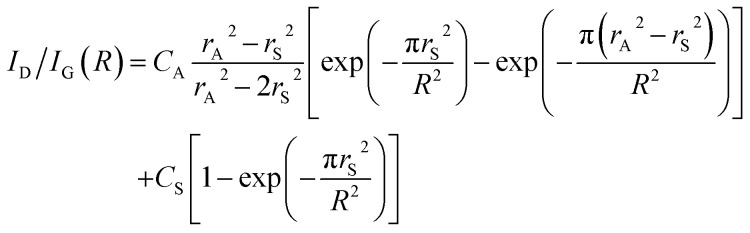
where *r*_S_ = 1.00 nm is the mean radius of the structurally disordered region and *r*_A_ = 3.00 nm is the mean radius of the activated region, where the graphitic structure is mostly conserved. However, its symmetry-breaking structure enhances the D band. *C*_A_ = 4.2 and *C*_S_ = 0.87 are parameters that describe the strength of the influences of the structurally disordered and activated regions on the intensity of the D band, respectively.^116^ Based on this equation, the mean density of vacancy defects per hexagon of the graphene framework was 10^−2^ for nanoporous carbons (NPCs) obtained by CH_4_-CVD and subsequent acid etching, whereas annealing of NPCs reduces the defect density to 10^−3^ to afford the corresponding NPGs without any loss of porosity.^86^

**Fig. 4:**
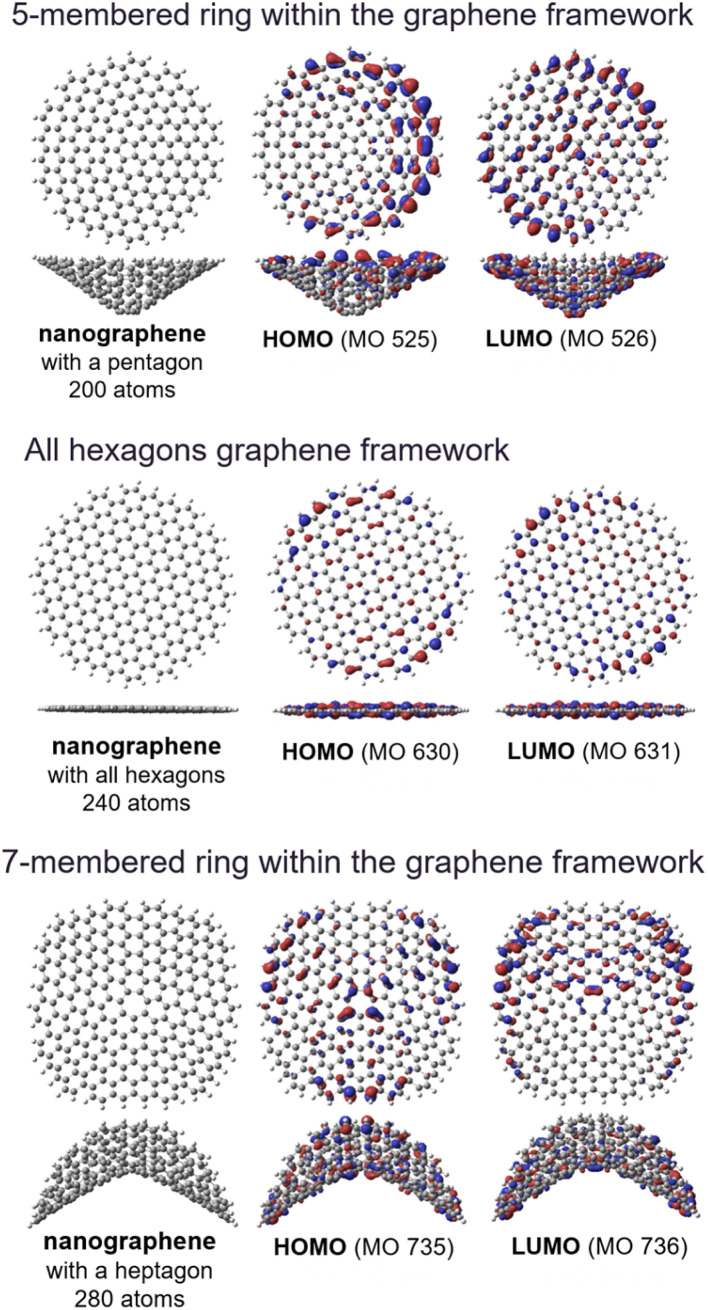
Optimized geometries of nanographene models, and the corresponding Kohn–Sham highest molecular orbital (HOMO) and lowest molecular orbital (LUMO) obtained using the DFT method.

The small peaks at ∼1150 cm^−1^ in the Raman spectra originate from heptagons and pentagons^5,113^ in the grain boundaries^117^ and surface defects^118,119^ within continuous graphene-based materials^59,92^ rather than edge sites. The domain boundary often contains 5- and 7-membered rings,^3–5,117–119^ which result in the curved structure shown in Fig. 4.

Fig. 4 shows the geometries and molecular orbitals of nanographene models having a pentagon and heptagon as a structural defect. Both the HOMOs and LUMOs of the structural models were relatively localized around the edges of the curved nanographene structures, and the excellent chemical and electrochemical stability of the NPGs can be explained by the reduced presence of reactive edges, as confirmed by TPD analysis.^92,100^

## 3. Application of ordered porous graphene materials

### 3.1 Electric double-layer capacitors (EDLCs)

**Fig. 5:**
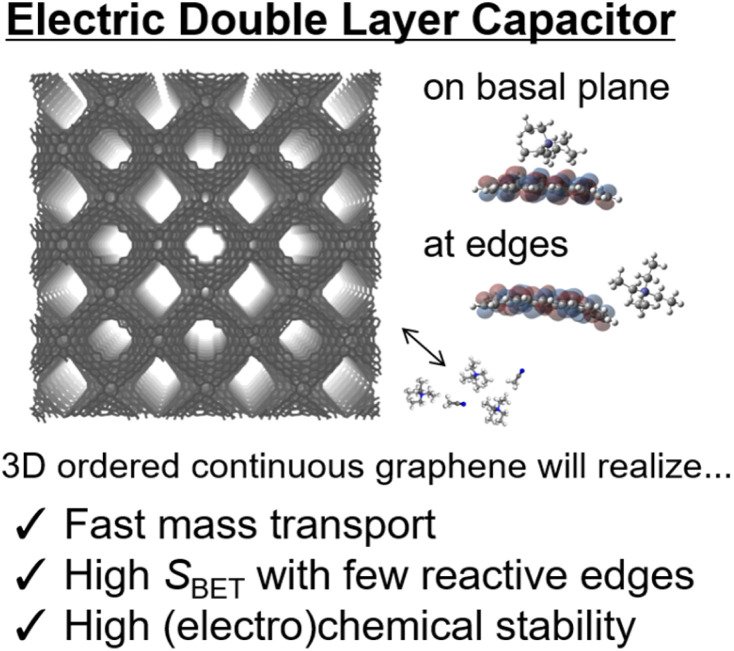
Schematic of electric double-layer capacitor (EDLC) application of high-quality 3D continuous graphene materials. Edge sites are electrochemically reactive,^39^ and therefore, NPGs with suppressed edges will be highly promising for EDLC applications.

The most common application of highly ordered nanoporous carbon materials is electric double-layer capacitors (EDLCs):^1,39,100^ EDLCs are extensively used in electric vehicles and other industrial fields. There are two important prerequisites for achieving efficient EDLCs. First, a high surface area of carbon materials is required to achieve EDLCs with high energy density, as *S*_g_ and *S*_v_ are directly related to the gravimetric and volumetric capacities, respectively.^120^ This can be realized by high porosity and small pore size because EDLCs can be charged in principle through the physisorption of electrolyte ions onto the nanopore surfaces of a carbon electrode.^120^ Importantly, this often causes a lower efficiency of mass transportation within nanostructured materials because the ions have to be transferred from the outside into the porous architecture of the electrode materials before physisorption in the EDLCs can occur (Fig. 5).^40^

3D structural regularity helps overcome these issues. For example, despite their microporous structure, ZTCs facilitate the efficient conveyance of ions under electrochemical conditions owing to their highly ordered architecture. This results in a high gravimetric capacitance of 140–190 F g^−1^ even at a high current of up to 20 A g^−1^.^39,121^ Ideal 3D graphene materials with such structural regularity and a continuous graphene architecture will realize highly efficient EDLCs in terms of energy efficiency and stability.

Liu and co-workers used ZTCs with different pore sizes as theoretical models to study the effect of pore geometry on capacitance.^122^ According to their study, the charge compensation per carbon (CCpC) determines the capacitance, and a high CCpC tends to be related to a small radius of curvature of graphene. Tang and co-workers used NPGs with different graphene-stacking layers (the specific surface area changed accordingly) to explore the influence of the specific surface area on the total capacitance and the origin of the total capacitance of 3D porous graphene.^123^

These results suggest that the origin of the capacitance is highly related to the specific surface area, pore structure, and surface chemistry of the carbon materials.^124–129^ The critical factor for obtaining carbon materials with large capacitance is the presence of fewer graphene-stacking layers in the carbon frameworks with appropriate surface modification to ensure a large electrical double-layer capacitance. The ordered and well-defined structures of ideal 3D graphene materials are particularly interesting in exploring the origin of capacitance and methods to improve them further.

### 3.2 Next-generation Li-ion batteries

Since their first introduction by the Sony Corporation in 1991,^130^ lithium-ion batteries (LIBs) have been highly important energy-storage techniques for use in mobile phones and electrical vehicles.^131^ For achieving the electrification of heavy-duty vehicles and aircraft and storing solar energy in smart grids, developing much better secondary batteries with much higher specific energy will be necessary.^132^ Air–metal batteries, including Li–O_2_ systems with Li-rich oxides as cathode materials, are promising candidates because of their high theoretical energy density.^133^ For practical application, the challenges of Li–O_2_ batteries are (i) handling of electronically insulating and insoluble Li_2_O_2_ as the discharge product, (ii) low discharge capacity of Li–O_2_ compared to theoretical capacity, and (iii) poor electrochemical cycling owing to chemical degradation.^134–142^ For better electrical conduction, graphene and its analogs have been frequently used as conductive additives in cathode composites.^140,143–146^ Ideally, 3D analogs of graphene materials would be suitable for achieving better electrical conductivity and supporting the reactants at the same time.

**Fig. 6:**
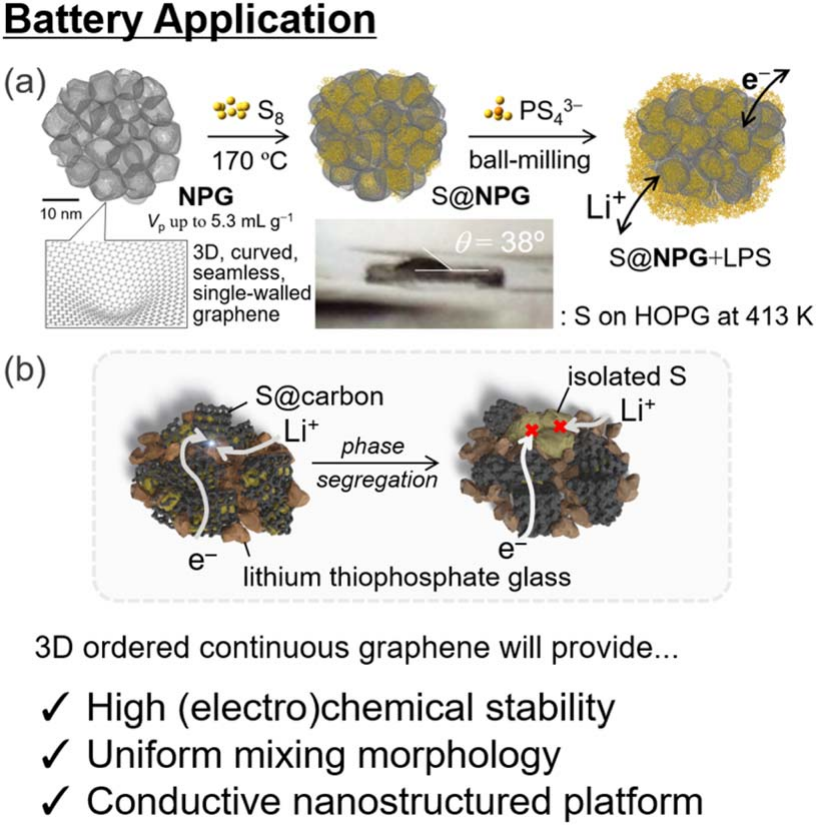
(a) Schematic of battery application of nanoporous graphene materials and (b) proposed degradation pathway which could be avoided by using the nano-confinement effect of NPGs^92^ (reprinted with permission from ref. 92. Copyright 2021 American Chemical Society). 3D-ordered continuous graphene provides a precise structure–activity relationship, which is conducive to better understanding and control of applications.

NPGs are promising scaffolds for novel LIBs because they have a high electron conductivity of up to 18 S cm^−1^ and excellent electrochemical stability owing to fewer reactive edge sites. NPGs also exhibit flexibility with an extremely low bulk modulus of <1 GPa. NPGs with large pore volumes and flexible nanopores have been applied as conductive additives in all-solid-state Li–S batteries,^92^ with better electrochemical performance than MSC30-based cells. The 3D continuous graphene structure in the NPGs annealed at 1800 °C under an inert atmosphere results in higher electrochemical stability and better cycling.^86^ In addition, the nano-confinement effect of insulating sulfur in conductive NPGs realizes durable three-phase contact (Fig. 6).^92^ This strategy can also be applied to other batteries with different active materials. Tailor-made 3D ordered nanopores with an arbitrary pore size and structure provide improved nano-confinement and clear-cut structure–activity relationships for better battery systems, including Li–O_2_ systems, when coupled with catalytic centers^102,147,148^ with better morphology for controlling cathode–electrolyte interaction^149^ and electrochemical cycling.

Powder-type NPGs have the potential problem of grain-boundary resistance and are difficult to handle. Pelletization of the template materials affords monolith-type NPGs after CH_4_-CVD, thereby improving the connection of the grown nanographenes.^150^

### 3.3 Refrigeration based on mechanical force

Efforts to achieve ideal 3D graphene materials would also be beneficial for high-efficiency refrigeration systems. The introduction of curvature into single-layered graphene substantially reduces Young's modulus (*Y*). For example, the aforementioned ZTC and NPGs have a bulk modulus that is three orders of magnitude smaller than that of pristine single-layered graphene (Table 1).

**Fig. 7:**
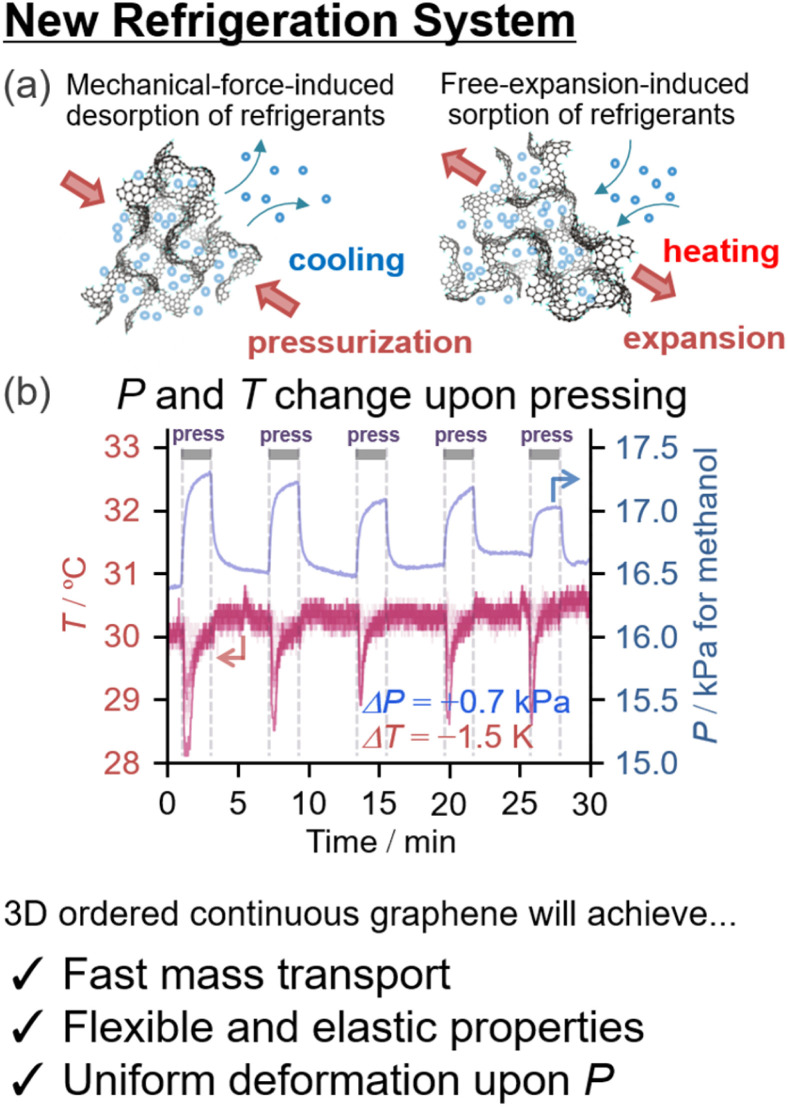
(a) Schematic of a new refrigeration system based on the flexible deformation of ordered nanoporous graphene materials^152^ upon pressurization,^99^ and (b) time-course of gas-phase pressure and temperature on the surface of the materials.^99^

We demonstrated that the nanopores of carbon materials could be reversibly deformed upon external pressurization up to 500 MPa and that the elastic properties are supported by quasi-linear stress–strain curves with nearly no loss in energy. In the presence of water or alcohol vapor, the gas–liquid equilibrium is significantly and compulsively altered upon deformation of the nanopores (Fig. 7a). This phenomenon could be used for refrigeration based on mechanical-force-induced phase transition of adsorbate (RMPTA) systems.^99^ NPGs with small *Y* values are a promising class of materials for RMPTA combined with water and alcohols as environmentally friendly adsorbates because the coefficient of performance (COP) is proportional to the reciprocal of the Young's modulus of the material (*Y*^−1^). Slow diffusion of molecules is a general issue related to the application of porous materials,^31,151^ but ideal 3D graphene materials with structural regularity can achieve fast diffusion of gases and a more flexible 3D continuous graphene structure simultaneously.

New ordered 3D NPG materials will realize better refrigeration systems based on the new operating principle with a uniform hierarchical structure (nm-to-mm scale) and resilience against deformation upon pressurization during the refrigeration cycle. A major impediment to efficient RMPTA systems is the uneven deformation of porous carbon materials under external pressurization. As shown in Fig. 7b, a sharp drop in temperature is observed during the compulsive liquid–gas phase transition upon pressurization, whereas nearly no heating is discernible despite the total recovery of the gas-phase pressure upon release. This irreversibility could be because of the lack of local structural robustness toward nonuniform stress. The uneven and irreversible structural changes in the nanopores are partly attributed to the structural heterogeneity of the NPGs owing to the inheritance of the irregular secondary particle structure appearing in their template materials, such as Al_2_O_3_ and zeolites. Another issue is the low thermal conductivity, and uniform nanographene growth in NPGs can also be achieved through improved CVD chemistry (Fig. 3b).

## 4. Conclusion and outlook

Since the proposal of Mackay crystals, several carbon materials have been reported to achieve high surface areas, structural regularity, and functionality. ZTCs are a class of carbon materials with a highly ordered 3D structure and a high specific surface area, while NPGs are extraordinary pluripotent materials with high electrochemical stability owing to fewer electrochemically active edges, a high electrical conductivity of up to 18 S cm^−1^ because of seamless and 3D-developed single graphene sheets, and highly flexible mechanical structure with a bulk modulus of less than 1 GPa. We investigated a general strategy for synthesizing high-quality and continuous 3D nanographene materials using CH_4_-CVD on nanosized oxide surfaces, where the least stacking of nanographene was achieved to ensure a high surface area with suppressed edge sites. Further investigation will expand the scope of template materials, including ordered oxides^101,103^ for synthesizing innovative 3D porous graphene materials in due course. However, challenges remain in achieving better homogeneity of nanographene growth and control of the hierarchical structure for better applications. As for 2D graphene growth,^153,154^ better control of CVD chemistry by suitable surface catalysis^101,155^ including well-controlled defects and dispersed metal-containing reactive sites coupled with computational chemistry^156,157^ will be crucial for better 3D graphene materials toward minimal surface graphene.^53,158^

**Fig. 8:**
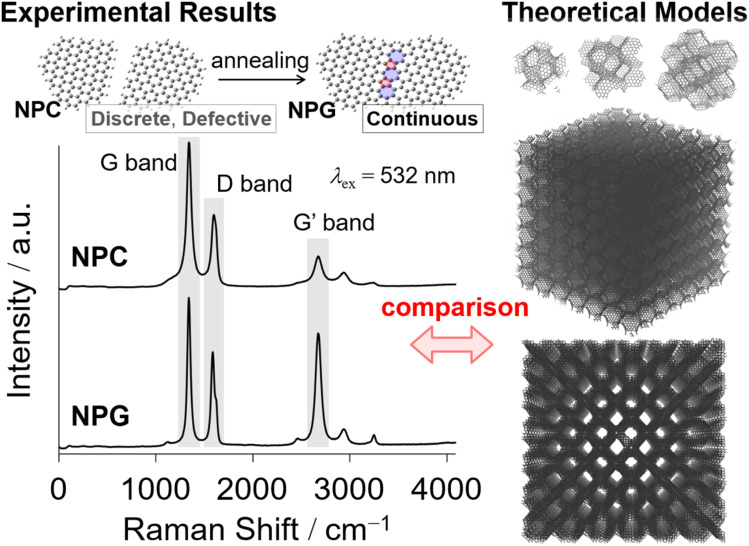
Schematic of the integration of experimental results and computational chemistry, and modeling for molecular understanding of the obtained ordered porous 3D graphene materials. Various spectroscopic techniques are of interest for elucidating the structure at the atomic level in a self-consistent manner, including Raman spectroscopy,^86^ angle-resolved photoemission spectroscopy,^23,170^ X-ray absorption fine structure spectroscopy,^171,172^ electron energy loss spectroscopy,^173^ X-ray photoelectron spectroscopy,^171^ steady-state absorption spectroscopy, and neutron diffraction.^174^

A comprehensive understanding of the structure of NPGs has been one of the central issues in this field. Recent progress in the advanced characterization of carbon materials has accelerated our understanding and control of the nanostructure of advanced carbon materials. We addressed the qualification and quantification of surface defects in NPGs by TPD^86^ and Raman spectroscopy,^159–161^ whereas Poisson statistics are useful for quantitatively determining the distribution of graphene layers^86,162–164^ in continuous 3D graphenes. The complementary and convincing direct observation of defects by electron microscopy techniques^3,165–167^ will result in a much deeper understanding of the nanostructure. These insights into the atomic level structure of NPGs including the chemical and topological defects will be conducive to further control of high-quality NPGs. Advanced characterization, including computational^168,169^ and experimental (Raman spectroscopy,^86^ angle-resolved photoemission spectroscopy,^23,170^ X-ray absorption fine structure spectroscopy,^171,172^ electron energy loss spectroscopy,^173^ X-ray photoelectron spectroscopy,^171^ steady-state absorption spectroscopy, and neutron diffraction^174^) approaches (Fig. 8), will provide a more sophisticated understanding and control of novel 3D graphene materials at the atomic level.

The successful application of NPGs and potential applications of ideal 3D graphene materials in supercapacitors, LIBs, and new refrigeration systems are also discussed in this review. Ideal 3D graphene materials will realize otherwise incompatible properties at the same time, namely, high electronic conductivity, ordered nanoporosity, flexibility, and high chemical and electrochemical stability. The realization of structural regularity of NPGs can produce these features simultaneously, and it is anticipated that such unprecedented carbon materials will have new unexplored applications, including catalyst supports in gas-phase catalysis (for example, CO_2_ hydrogenation^175^ and CH_4_ activation^73,78^) as “hydrophobic analogs” of zeolites. A systematic investigation of tuned pore sizes^122,141^ could also be considerably interesting for identifying otherwise hidden scientific aspects for many applications, including catalysis and batteries. Mechanical-force-induced changes in the nanopores of current NPGs (Fig. 7) occur nonuniformly because of the uneven structure of current carbon materials. Therefore, continued efforts to create ordered porous graphene materials with uniform hierarchical structures from the micro- to macro-scale will help improve the efficiency of these applications. The combination of innovative synthesis, advanced characterization, and unexplored applications will open a new route for achieving more energy-efficient systems using next-generation NPGs.

## Data availability

All data associated with this study will be available upon request to the authors.

## Author contributions

The manuscript was written with the contributions of all authors, and all authors approved the final version.

## Conflicts of interest

There are no conflicts to declare.

## Supplementary Material
